# *Crocus* *sativus* (Saffron): A potential multifunctional therapeutic agent for neurodegenerative disorders

**DOI:** 10.7555/JBR.38.20240131

**Published:** 2024-08-16

**Authors:** Mohammad Reza Khazdair

**Affiliations:** Pharmaceutical Science and Clinical Physiology, Cardiovascular Diseases Research Center, Birjand University of Medical Sciences, Birjand 9717853111, Iran

Dear Editor,

Neurodegenerative disorders (NDDs) are age-related disorders characterized by the deposition of abnormal forms of proteins or the progressive loss of neurons in the brain. NDDs are classified according to their pathophysiological properties, including cognitive dysfunction, Alzheimer's disease (AD) and other dementias, Parkinson's disease, and motor neuron disease. The increased oxidative stress and neuro-inflammatory events contribute to neuronal atrophy and death in NDDs^[1]^. Nitric oxide in the central nervous system has several functions, including the regulation of synaptic plasticity and neurosecretion. The physiological amount of nitric oxide is neuroprotective, whereas higher doses are noticeably neurotoxic^[2]^.

The nuclear factor erythroid 2-related factor 2 (NRF2), the main regulator of redox status, regulates cellular oxidative and inflammatory balance by interacting with the nuclear factor kappa-light-chain-enhancer of activated B cells (NF-κB)^[3]^. In hippocampal neurons from AD brains, NRF2 is principally localized in the cytoplasm rather than in the nucleus, whereas in the normal human hippocampus, NRF2 protein is detected in both the nucleus and the cytoplasm of neurons^[4]^.

*Crocus* *sativus* L. (Iridaceae), or saffron, is widely cultivated in Asia and Spain, and it has been used as a dietary spice, food additive, and for various medicinal purposes in Iranian traditional medicine^[5]^. Saffron suppresses the activation or production of a protein complex (NF-κB) that controls DNA transcription, cytokine production, and cell survival. Saffron and its ingredients also inhibit inflammatory enzymes, such as cyclooxygenase-2, myeloperoxidase, and inducible nitric oxide synthase, playing a critical role in the pathophysiology of diseases^[6]^. Saffron and its active constituents exhibit neuroprotective effects through several mechanisms, such as inhibiting amyloid-β (Aβ) aggregation and deposition in AD, protecting dopaminergic cells, modulating the cholinergic system, increasing levels of glutathione and its dependent enzyme, and suppressing the increase in malondialdehyde (MDA), glutamate, and aspartate levels^[7]^.

The administration of safranal, a saffron constituent, was found to significantly attenuate rotenone-induced cell death in dopaminergic neurons and restore the protein expression levels of B-cell lymphoma 2 (BCL2) and BCL2-associated X protein (BAX); in addition, the pretreatment of the cells with safranal markedly decreased the expression levels of Kelch-like ECH-associated protein 1 (KEAP1) and upregulated the expression of nuclear NRF2 that changed by rotenone^[8]^. These data suggest that safranal has a protective effect against rotenone-induced cell death in dopaminergic neurons.

The results of a systematic review of clinical trials (SRCT) indicated that saffron might be an antidepressant because of its serotonergic, anti-inflammatory, antioxidant, and neuroprotective effects, similar to the prescribed antidepressant drugs^[9]^. Another systematic review and meta-analysis also showed that saffron possessed a better efficacy for improving depressive symptoms, compared with the placebo treatment, and it was as effective as synthetic antidepressants; furthermore, the incidence of adverse effects between saffron and antidepressants was not significantly different^[10]^. One systematic review of randomized controlled trials (RCTs) with 325 individuals showed that saffron was well-tolerated and significantly improved scores on the AD Assessment Scale-Cognitive Subscale (ADAS-cog) or Mini-Mental State Examination, compared with placebo, and that these effects did not differ significantly, compared with donepezil or memantine^[11]^.

Similarly, one systematic review and meta-analysis of RCTs also revealed that saffron significantly improved cognitive function measured by the ADAS-cog, compared with the placebo groups; furthermore, the cognitive scales such as ADAS-cog were not significantly different between saffron and conventional medicine treatment^[12]^. Saffron supplementation in unhealthy patients in RCTs significantly reduced MDA levels and concurrently increased the total antioxidant capacity^[13]^. The results of another systematic review and meta-analysis in RCTs also indicated that saffron caused a significant reduction in MDA and total oxidant status levels, but a significant increase in the total antioxidant capacity and glutathione peroxidase levels. Moreover, the subgroup analysis indicated a significant reduction in MDA levels in those aged < 50 years, with saffron dosage of > 30 mg/day, and study durations of < 12 weeks^[14]^. The characteristics of the included SRCT studies are summarized in ***Table 1***.

**Table 1:** Characteristics of the included studies

References	Location	Study population	Sample size	Number of studies	*Crocus* *sativus* dosage	Study duration (weeks)	Main outcome
Lopresti *et* *al*, 2014^[9]^	Iran	Depression	230	6	30 mg/day delivered in two equal doses	6	Showing large treatment effects compared with a placebo and was as effective as the antidepressants (fluoxetine and imipramine).
Dai *et* *al*, 2020^[10]^	Iran	Depression	612	12	30 mg/day in 11 studies and 50 mg/day in one study	6 to 12	Saffron was effective for treating mild to moderate depression and had comparable efficacy to antidepressant drugs.
Avgerinos *et* *al*, 2020^[11]^	Iran and Greece	Cognitive function	325	5	10 to 30 mg/day	12 to 48	Showing similar efficacy in improving cognitive scores as common anti-Alzheimer's disease drugs.
Ayati *et* *al*, 2020^[12]^	Iran and Greece	Cognitive function	203	4	30 mg/day	16 to 48	Saffron showed beneficial effects in improving cognitive function in patients with Alzheimer's disease and mild cognitive impairment.
Morvaridzadeh *et* *al*, 2021^[13]^	Iran	Oxidative stress parameters	651	10	30 mg/day in five studies, 100 mg/day in four studies, and 1000 mg/day in one study	4 to 12	Saffron intake significantly decreased malondialdehyde levels but significantly increased total antioxidant capacity. Saffron showed beneficial properties in improving overall survival in unhealthy patients.
Abedi *et* *al*, 2023^[14]^	Iran	Oxidative stress parameters	468	16	30 to 100 mg/day	4 to 12	Saffron and its active ingredients were able to establish a balance of oxidants/antioxidants in various disease conditions in trial studies.

Results of the reviewed studies have indicated that the consumption of saffron and its constituents in both basic and clinical studies are capable of having significant properties on memory and cognitive deficiency through cell signaling pathways, such as the modulation of inflammatory and pro-inflammatory mediators and clearance of Aβ aggregation. Treatment with saffron significantly represses the generation of ROS, oxidative stress, and cell apoptosis in *in* *vitro* studies.

Results from *in* *vivo* studies have shown that saffron and its constituents exhibit neuroprotective effects through various mechanisms, such as modulating neurotransmitters, enhancing neurogenesis, reducing neuro-inflammation, regulating oxidative stress, activating the NRF2 signaling pathway, and modulating epigenetic factors^[15–16]^. Several clinical and preclinical studies have also demonstrated the efficacy and safety of saffron as well as its constituents in improving cognitive function, mood, and other neurological outcomes^[17–18]^. Although the exact mechanisms for the neuroprotection of saffron are not explicitly known, the results of several systematic reviews and meta-analyses indicated that saffron showed beneficial effects on NDDs through various different mechanisms, including cell signaling pathways, modulation of pro-inflammatory mediators, and inhibition of oxidative events. Thus, regarding the safety and efficacy of saffron as a food additive, it may be considered a complementary therapy for reducing or treating NDDs.

Yours Sincerely,Mohammad Reza Khazdair^✉^ Pharmaceutical Science and Clinical Physiology, Cardiovascular Diseases Research Center, Birjand University of Medical Sciences, Birjand, South Khorasan 9717853111, Iran.^✉^Corresponding author: Mohammad Reza Khazdair. E-mail: M.khazdair@yahoo.com.
